# Lipid Biomarkers in Urban Soils of the Alluvial Area near Sava River, Belgrade, Serbia

**DOI:** 10.3390/molecules30010154

**Published:** 2025-01-03

**Authors:** Gordana Dević, Sandra Bulatović, Jelena Avdalović, Nenad Marić, Jelena Milić, Mila Ilić, Tatjana Šolević Knudsen

**Affiliations:** 1 Institute of Chemistry, Technology and Metallurgy, National Institute of the Republic of Serbia, University of Belgrade, Njegoševa 12, 11000 Belgrade, Serbia; sandra.bulatovic@ihtm.bg.ac.rs (S.B.); javdalovic@chem.bg.ac.rs (J.A.); jelenamilic@chem.bg.ac.rs (J.M.); milailic@chem.bg.ac.rs (M.I.); tsolevic@chem.bg.ac.rs (T.Š.K.); 2 Faculty of Forestry, University of Belgrade, Kneza Višeslava 1, 11030 Belgrade, Serbia; nenad.maric@sfb.bg.ac.rs

**Keywords:** soil, petroleum pollution, lipid biomarkers, *n*-alkanes, evaluation indices, hierarchical cluster analysis (HCA)

## Abstract

This study focused on the investigation of soil samples from the alluvial zone of the Sava River, located near the heating plant in New Belgrade, Serbia. Using gas chromatography with flame ionization detection (GC-FID), a broad range of alkanes, including linear *n*-alkanes (C_10_ to C_33_) and isoprenoids, was analyzed in all samples. The obtained datasets were effectively made simpler by applying multivariate statistical analysis. Various geochemical indices (CPI, ACL, AI, TAR, etc.) and ratios (S/L, Paq, Pwax, etc.) were calculated and used to distinguish between biogenic and anthropogenic contributions. This approach added a higher level of precision to the source identification of hydrocarbons and provided a detailed geochemical characterization of the investigated soil. The results showed that the topsoil had a high content of TPH (average value, 90.65 mg kg^−1^), potentially related to an accidental oil spill that occurred repeatedly over extended periods. The uncommon *n*-alkane profiles reported for the investigated soil samples are probably the result of inputs related to anthropogenic sources, emphasizing that petroleum was the main source of the short-chain *n*-alkanes. The methodology developed in this study was proven to be efficient for the assessment of the environmental quality of the soil in an urban part of New Belgrade, but it can also be a useful tool for soil monitoring and for a pollution assessment in other (sub)urban areas.

## 1. Introduction

The primary sources of soil contamination in metropolitan areas are anthropogenic activities. Petroleum products are among the most frequently found pollutants of urban soils. In populated areas, they can be generated from both point and diffuse sources, and differentiation of contributions from different sources is possible with the application of instrumental techniques such as gas chromatography with flame ionization detection (GC-FID) and gas chromatography–mass spectrometry (GC-MS) [1]. However, even with the great progress made in the field of environmental forensics in recent decades, elucidating the origin and the fate of organic compounds in the environment continues to be a challenging and highly demanding task [2].

The sources of petroleum hydrocarbons in urban soils are diverse, and they include traffic, production and usage of refined and crude oils, dust and airborne particles from factories, incinerators, hydrocarbon-fueled power plants, etc. [3]. One group of petroleum hydrocarbons that is present in the environment in significant concentrations is the group of aliphatic hydrocarbons (AHs). Previous studies have confirmed that the AHs in the environment can originate from a wide range of biogenic and petrogenic sources [4]. Terrestrial vascular plants, planktons, bacteria, algae, and plant and animal biomass are the primary natural sources of AHs, whereas crude oil and its derivatives are the main anthropogenic sources of these compounds in the environment [5]. Due to their low water solubility and to their hydrophobic characteristics, AHs are able to aggregate, settle, and accumulate as suspended particles in soils and sediments [6]. As a result, urban soils and roadway dust can serve as a conduit for the transfer of these pollutants into runoff and for their subsequent accumulation in soils and sediments [7].

The source of petroleum pollutants in environmental systems directly determines the hydrocarbon content, particularly the content and distribution of *n*-alkane compounds [8]. Some constituents of the petroleum pollutants are able to retain the signatures of their sources, and because of that, they are referred to as geochemical, petroleum, or molecular (bio)markers [9,10,11,12]. Petroleum biomarkers are stable molecules that are able to withstand transformations in the environment, and therefore, they can be used as indicators that can confirm the presence of hydrocarbons with anthropogenic origin [13]. *n*-Alkanes and isoprenoids pristane and phytane are lipid biomarkers that are traditionally used to distinguish between biogenic and anthropogenic inputs of AHs into the environment [14,15,16,17]. In environmental geochemistry, distributions and abundances of *n*-alkanes and isoprenoids are usually used to calculate typical evaluation indices and diagnostic ratios. Among them, the most commonly used are the carbon preference index (CPI), the terrigenous/aquatic ratio (TAR), proxy ratios (Paq and Pwax), average chain length (ACL), total petroleum hydrocarbons (TPH), unresolved complex mixture (UCM) in relation to the resolved alkanes (U/R), and the proportion of short to long *n*-alkanes (S/L).

The aim of the present study was to determine the origin and the pollution levels of petroleum hydrocarbons in urban soils within the alluvial deposits of the Sava River, close to the heating plant in New Belgrade, Serbia. For that purpose, the content of total petroleum hydrocarbons (TPHs), the determination of biomarkers, *n*-alkanes, and different evaluation indices (CPI, ACL, TAR, Paq, Pwax, L/H alkanes, UCM, U/R, Pr/Phy, pristane/*n*-C17, and phytane/*n*-C18) were employed. These parameters were also used in this study to differentiate between the multiple origins of AHs. Multivariate statistical techniques, which provide a strong tool for data reduction and geochemical data interpretation, were applied in this study to discover the underlying causes of variance in the soil geochemistry [18].

The study site represents the extensive alluvial zone of the Sava River, with an intergranular aquifer. The heating plant is situated on the Sava River’s left bank, within a residential and traffic area of New Belgrade, which is home to over 200.000 residents [19]. In previous decades, the heating plant relied on the extensive usage of fossil fuels. Because of that, this plant represents a potential source of petroleum products in this part of the alluvial area of the Sava River and, therefore, has been selected as a study area for the current research. This presented study introduces a method that combines the research of the sources of petroleum pollutants found in the study area’s surface soil and a statistical analysis. The methodology applied involves identification of lipid molecular biomarkers, determination of various evaluation indices that aim to define the origin of these compounds, and usage of multivariate statistical analysis to facilitate data interpretation. Although this investigation is focused on this specific area, more broadly, this methodology can be applied to assess potential urban impacts in other similar hydrogeological settings.

## 2. Materials and Methods

### 2.1. Study Area

The study area is located near one of the largest heating plants in SE Europe (New Belgrade, Serbia). The investigated site is defined by the coordinates E 20°24′22.50′′–20°24′47.46′′ and N 44°47′53.04′′–44°48′10.26′′, and it is at the altitude of 120 m above sea level.

**Figure 1:**
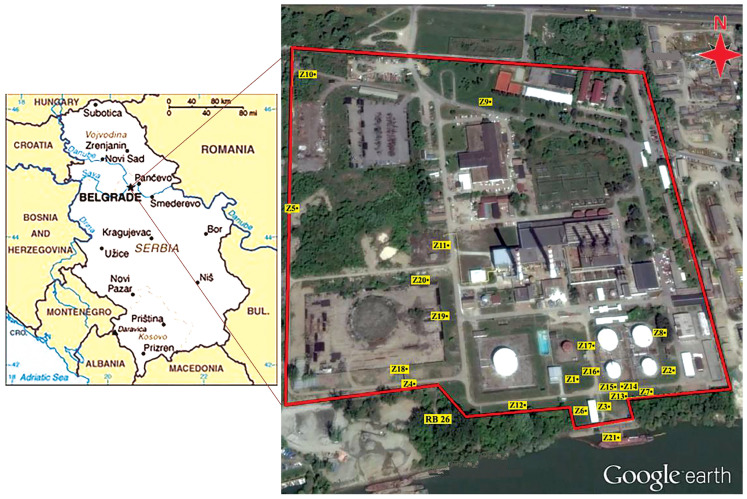
Investigated area—the heating plant in New Belgrade, Serbia, and the positions of sampling microlocations.

According to geological and hydrological characteristics, the area investigated in this study is situated in the alluvial plain of the Sava River (Figure 1). Within this part of the alluvial plain, there is a network of radial collector wells used for groundwater extraction and urban water supply. Extensive research at this locality was conducted between 2015 and 2019, which included analyses of soil and water, as well as of water samples from the Sava River [19,20,21]. Those previous studies were focused on the investigation of potentially toxic elements in the urban sediments at this locality [19] and on the bioremediation of groundwater contaminated with petroleum hydrocarbons in this area [20,21]. The aim of our present study is to determine the origin and the pollution levels of petroleum hydrocarbons in soil at this locality. Since the regional contamination patterns are unknown, the study focused on local hotspots to identify and analyze potential sources of hydrocarbon contamination that can have a wider significance and to support the establishment of regional databases with the contamination patterns of the specific environmental pollutants.

### 2.2. Sampling and Analytical Strategy

In order to determine the extent and the severity of contamination by oil pollutants, as well as the origin of these contaminants at the investigated location, a sampling and analytical strategy was developed to include the following steps: preliminary site assessment; development of a sampling plan; development of a soil sampling methodology; development of an analytical methodology; quality assurance and quality control (QA/QC); and development of a methodology for data interpretation.

The preliminary site assessment included review of the historical data related to the sampling location. Specific information was collected about the site activities, historical petroleum spills, previous analytical campaigns, and previous remediation efforts. Visual inspection was used during the on-site visits in order to identify areas with visible signs of contamination. Preliminary hazard identification was conducted with the aim of evaluating possible risks associated with exposure to the set of pollutants present at this location.

A specific sampling plan was used to identify the sampling locations. Considering the focus of the current study, the surface soil was recognized as the main target for this research. Because of that, the sampling depth was set to be 0–30 cm, and 20 sampling points were carefully selected to appropriately represent the study area. The sampling points within the study area are shown in Figure 1. All details related to the soil sampling methodology are explained in the Supplementary Material.

An appropriate analytical methodology was developed to ensure the employment of the appropriate methods for determination of the AHs, which are the main focus of this study. To this aim, total petroleum hydrocarbons (TPH) were determined according to the standard methods, BS EN ISO 16703 (2011) and DIN EN 14345 (2008) [22,23]. The total solvent extractable organic substance from the analyzed soil samples was extracted for 36 h with dichloromethane as a solvent, using a Soxhlet method. These extracts were fractionated by liquid chromatography into saturated hydrocarbons (Fraction I), aromatic hydrocarbons (Fraction II), and polar compounds, alcohols and keto compounds (Fraction III), using a column with alumina and silica gel. For analysis of AHs, a GC-FID instrument was used (Agilent 7890A gas chromatograph with flame ionization detector, Agilent, Santa Clara, CA, USA). All analytical methods are detailed in the Supplementary Material.

A rigorous quality assurance and quality control (QA/QC) was followed during the GC-FID analyses. The instrument was calibrated regularly using the calibration standards. Instrumental performances were tracked according to the instrument quality control procedures. Laboratory, solvent, and procedural blanks were periodically included with each batch of samples (10 samples per batch) to assess a potential contamination, and the values were consistently below the detection limit [24]. All parameters of the instrumental analysis are detailed in the Supplementary Material.

### 2.3. Multivariate Statistical Analysis

Multivariate statistical techniques, including cluster analysis (CA) and correlation analysis, were applied to process the analytical data using SPSS software (version 22.0) for Windows (IBM Corporation, Armonk, NY, USA).

As part of the statistical data processing, multivariate statistical techniques were applied to the previously systematized geochemical parameters dataset. The data used for this purpose were composed from the diagnostic ratios based on the concentrations of AHs that were quantified in the soil samples collected in the urban area of Belgrade that was investigated in this study. Based on commonalities, the soil samples were grouped using hierarchical cluster analysis (HCA). Geochemical research can benefit from this methodology, and using cluster analysis, samples can be classified according to a wide range of characteristics [19,25]. In order to confirm the HCA results and to identify the variances in the variables that can be explained by their interactions with each other, Pearson’s correlation coefficient analysis (PCA) was also used [26].

## 3. Results and Discussion

### 3.1. Concentrations and Geochemical Characteristics of AHs in Soil

The contents of the extracted soluble organic substance, aliphatic hydrocarbons, aromatic hydrocarbons, asphaltenes, and polar NSO compounds are shown in Table 1.

The extracted soluble organic matter (SOM) content in the samples was found to be in the range of 102.59 to 1388.03 mg/kg (Table 1). The ratios of groups of compounds such as NSO, aromatic, and saturated are given in Table 1. The SOM of the analyzed soil samples consisted primarily of asphaltenes and polar NSO compounds (more than 50 wt.%), with a minimal amount of *n*-alkanes (with an average concentration of 5.13 wt.%). The ranges and means of the extractable groups of compounds in the surface soil samples analyzed in this study were compared with similar data from other towns in the world (Krakow, Tehran, and Riyadh metropolitan) in order to evaluate the soil quality of this study area. Generally, compared to Belgrade, the concentrations of aliphatic hydrocarbons in other urban areas are similar or much higher. However, it should be noted that comparisons between these studies are relative and that the reported values are all based on the methodologies used in the particular studies. As can be seen from Table 1, in the analyzed soil samples, two borderline cases can be noticed. The sample Z-16 has the highest amount of the extracted organic substance, but the smallest proportion of Fraction I. The sample Z-4 has the lowest concentration of the extracted organic substance, and a mean content of Fraction I, compared to the other two fractions. In the other analyzed samples, the content of Fraction I was much lower in comparison to the other fractions.

Due to the specific distribution pattern of *n*-alkanes in different organisms, these compounds can be useful markers that can be used to distinguish the biomass contribution of individual species of living organisms to the sedimentary OM of soils. The primary sources of biogenic *n*-alkanes in soils are cuticular waxes of plant leaves and roots. Land plants contain a high proportion of higher MW *n*-alkanes (C_27_, C_29_, and C_31_) in their epicuticular wax. Deciduous trees typically maximize at C_27_, whereas C_31_ is dominant in marsh plants and possibly grasses. Aquatic organisms can also contribute to the OM of soils, especially in the areas close to water bodies and in areas that are frequently exposed to flooding. Phytoplankton’s and algae are dominated by low-molecular-weight *n*-alkanes, maximizing at C_17_. Submerged/floating macrophytes maximize at C_21_, C_23_, and C_25_, while emergent macrophytes have a composition similar to that of terrestrial plants, peaking at C_27_ and C_29_. However, data from a broad survey of modern plants show that *n*-alkane chain-length distributions can be highly variable within plant groups and that it might be difficult to make chemotaxonomic distinctions between grasses and woody plants, with the exception of aquatic plants and sphagnum moss. In contrast, changes in chain length distribution are likely to be a result of temperature and/or humidity conditions [27,28,29,30,31,32,33]. Anthropogenic sources can also contribute to the *n*-alkane contents in soils. Fossil fuels and their derivatives are common anthropogenic sources of *n*-alkanes. A large proportion of molecules with short carbon chains (less than C_20_) and with similar abundances of odd and even homologs represent *n*-alkanes from anthropogenic sources [34,35]. According to Yang et al. [36], the presence of C_10_ in the chromatograms may also be a sign of recent contamination with light-distillated products such as gasoline or diesel.

**Figure 2:**
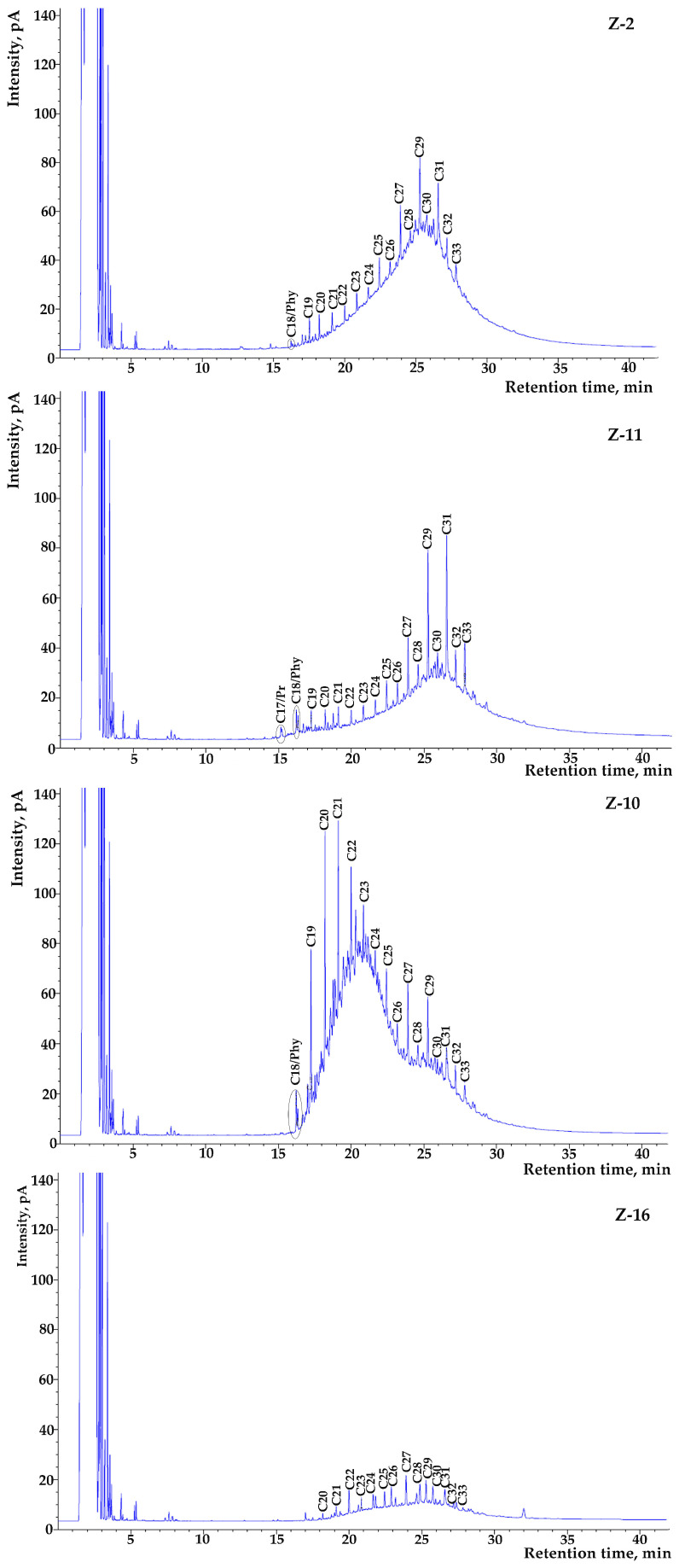
GC-FID chromatograms of *n*-alkanes in some soil samples from the alluvial area of New Belgrade.

Figure 2 shows some of the characteristic *n*-alkane profiles determined in the investigated soil samples of the present study. The common feature of the *n*-alkane profiles for these soil samples is the odd-number *n*-alkane predominance in the range from C_10_ to C_33_, with a significant maximization at C_29_ and/or C_31_ (characteristic of terrestrial plant wax inputs). The overall distribution pattern of the *n*-alkanes is common for all analyzed samples, except from sample Z-1. This sample exhibits a bimodal distribution pattern with two distinct parts. The first part is similar to the other samples, while the second one is in the *n*-alkane range from C_18_ to C_25_, maximizing at C_20_, indicating contributions from submerged floating macrophytes. In general, high levels of *n*-alkanes typically show the combined influence of petrogenic and biogenic origins in urban soils near the alluvial area of the Sava River in New Belgrade. The peak of C_18_ is notable, but it remains lower than the peak of C_17_ in all samples. In addition, in the Z-10 sample, the second-highest peaks belong to C_18_, C_19_, C_20_, C_21_, and C_22_, which are also related to petrogenic sources [8]. The presence of isoprenoids pristane and phytane in the investigated samples also confirms petroleum-based sources of AHs in these soils.

In the obtained GC/FID chromatograms (Figure 2), in addition to the identification of *n*-alkane profiles, a specific characteristic can be noticed. The peak resolution baseline in the GC-FID chromatograms is separated from the solvent baseline by a hump region, named the unresolved complex mixture (UCM), which is considered indicative of the environmental residuals from oil pollutants [37]. This appearance of the chromatograms originates from a complex mixture of branched and cyclic hydrocarbons that exhibit higher resistance to biodegradation compared to *n*-alkanes, and because of that, they persist and accumulate in the environment [38]. These compounds usually remain unresolved under capillary chromatographic conditions and form a recognizable hump or UCM in the chromatograms. For the samples analyzed in this study, the UCM covers a wide range of 2.97 to 78.38 mg/kg (Table 2), suggesting a high content of petroleum hydrocarbons in some of the analyzed soil samples.

The U/R ratio values range from 0.18 to 6.51 (Table 2). The samples Z-2, Z-3, and Z-9 have U/R values ≥ 4 (Table 2), indicating a high content of biodegraded petroleum-derived substances in these samples. These results indicate some earlier petroleum inputs into the related soil. Relatively low values of UCM for some samples may suggest recent inputs of the petroleum pollutants [38], as is the case with soils Z-5, Z-6, Z-11, and Z-12.

Although the UCM of oil pollutants in GC-FID chromatograms remains unexplored, attempts to resolve it are published in the scientific literature. For example, a comprehensive two-dimensional gas chromatography (GC×GC) was used in the manuscript by Frysinger et al., 2003 [39], to investigate the chemical composition of the unresolved complex mixture (UCM) of hydrocarbons in petroleum-contaminated marine sediments. The applied technique is important to resolve thousands of individual chemical components from the UCM and to understand the sources, weathering, and toxicity of UCM hydrocarbons. Accordingly, the future research of the samples analyzed in this study should include attempts to analyze these unresolved compounds with the aim of assessing their possible usage in the analysis of the contamination sources and in the analysis of possible toxicities of UCM hydrocarbons at this location.

In many countries and regions, the TPH level is one of the key factors in the determination of the ecologic status of soil. The content of TPH in uncontaminated areas is typically lower than 50 mg/kg of dry soil [40], while values above 100 mg/kg are considered an indicator of soil pollution with petroleum pollutants [41]. In the soil samples analyzed in the present study, the TPH concentrations ranged from 49.6 to 244 mg/kg, with a mean value of 83.7 ± 9.0 mg/kg (Table 2). The results showed that the topsoil at some sampling points had TPH levels higher than the threshold values of 100 mg/kg (102 mg/kg, 109 mg/kg, and 244 mg/kg at the sampling points Z-20, Z-19, and Z-18, respectively; Table 2). Accordingly, these results can be connected to accidental spills and leaks of petroleum or its derivatives at these three microlocations. The TPH level was lower than 50 mg/kg at only one sampling point (49.6 mg/kg at the sampling point Z-3; Table 2), implying uncontaminated status of the soil at this microlocation. For most of the analyzed samples, the results showed TPH levels between the values for uncontaminated soils (50 mg/kg of dry soil) and for soils contaminated with petroleum pollutants (100 mg/kg of dry soil; Table 2). According to these results, it can be presumed that most of the soil samples analyzed in this study contain a mixture of anthropogenic (petrogenic) and biogenic (terrestrial) organic matter.

In comparison with the TPH values reported for the topsoil’s in some other large cities, it can be concluded that the TPH values measured in our current study are significantly higher than those measured in some other cities (China: 11.40–21.50 μg/g, Turkey: 48.30 μg/g, Nigeria: 11–402 μg/g, and Indonesia: 52.33–159.94 μg/g), but they are also much lower than concentrations of TPH in London topsoil (0 to 2 cm topsoil with TPH ranged from 72 to 4673 mg/kg) [42].

Based on the remediation criteria established in the Republic of Serbia, it can be concluded that the TPH values reported in the present study are higher than the national remediation values [43]. These results necessitate remediation of the investigated soil and removal of petroleum hydrocarbon pollutants. Moreover, it should be emphasized that the contaminated soil is situated close to two radial collector wells of the Belgrade groundwater source for the city water system supply. This fact emphasizes the importance of the appropriate characterization of the contamination origin and spatial distribution, thus providing the basis for the appropriate selection and application of the conservation practices.

**Figure 3:**
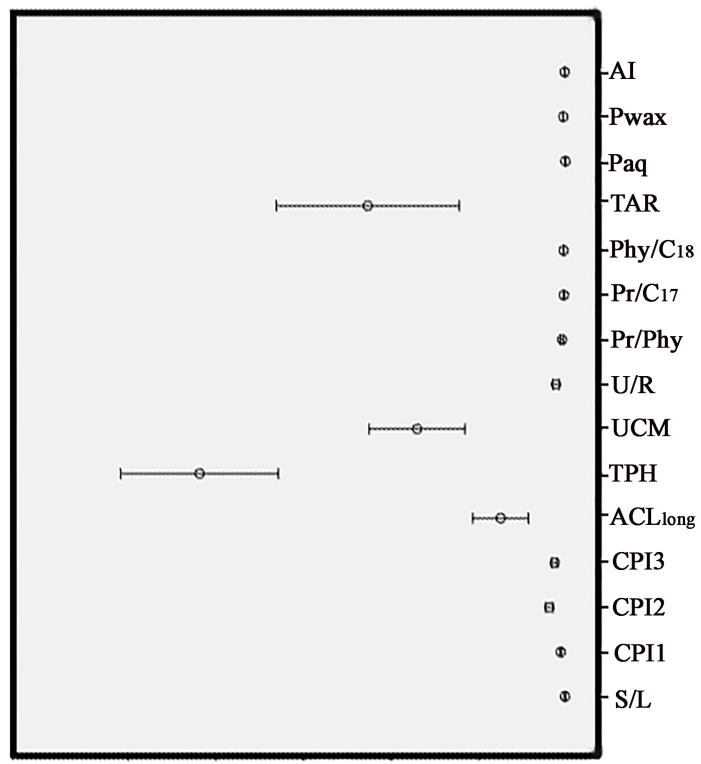
Box and whisker plot showing the variation of geochemical indices in the investigated soil samples.

The box and whisker plot analysis were used to represent the profiles of the variables of the soil samples, as shown in Figure 3. Parameters such as the TPH, UCM, TAR, and ACL indices exhibited a broad range as indicated by the high standard deviation values for these parameters.

### 3.2. Characterization of the Soil Samples According to the Organic Geochemical Indices

Live organisms typically synthesize a broad range of *n*-alkanes, most often with a strong odd-over-even predominance [14]. However, there are some literature cases of some live organisms, more specifically some algae, that are able to synthesize *n*-alkanes with a predominance of even homologues [16]. The carbon preference index (CPI) is a valid diagnostic tool based on the distribution of odd and even *n*-alkanes in samples. As such, it has been traditionally used for distinguishing between contamination from fossil fuels and from biogenic hydrocarbon sources. The usage of CPI for these diagnostic evaluations is based on the fact that *n*-alkanes from biogenic sources result in CPI values greater than 1 (due to the odd-over-even predominance), and for higher plants, they are in the range from 6 to 10. *n*-Alkanes in fossil fuels are characterized by a uniform distribution of odd and even homologues, and therefore, *n*-alkanes from crude oils and their derivatives have CPI values of ≈1.

The CPI values in the present study were found to be in the range of 1.28 to 6.64 (calculated for the entire *n*-alkane range, CPI 3; Table 2), with an average CPI value of 2.76 ± 1.43. These values are higher than the CPI values from inputs connected to fossil fuels, but they are lower than the values usually obtained from continental plant inputs. Because of that, it can be concluded that the OM in the soil samples investigated represents a mixture where fossil *n*-alkane series were mixed in different ratios with a higher land plant *n*-alkane series. The results for the samples Z-8, Z-15, Z-16, and Z-17 (all samples near petroleum tanks and the Sava River; Figure 1) with CPI values of around 1 confirm the presence of petroleum pollution. Higher CPI values obtained for the other analyzed soil samples indicate the domination of biogenic inputs to these samples.

**Figure 4:**
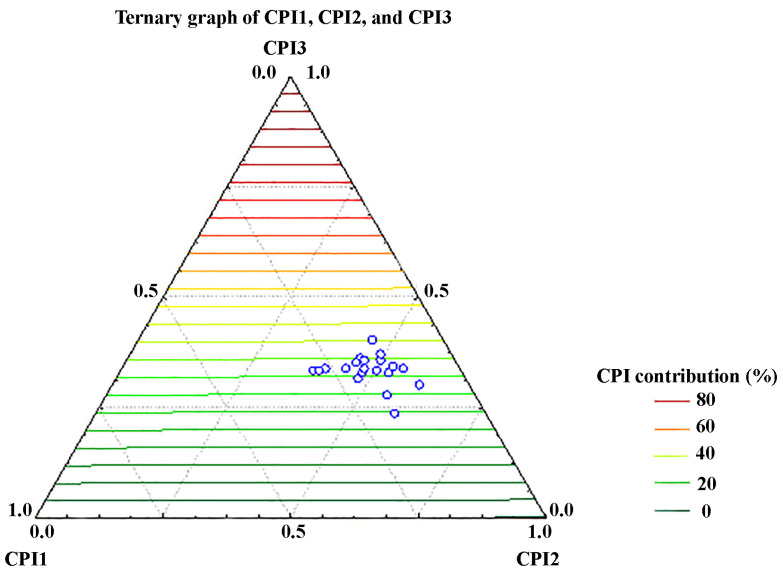
Ternary diagram showing relative abundance of the carbon preference indices: short-chain alkane homologs, CPI1, mid-chain *n*-alkanes, CPI2, and all *n*-alkanes homologs (CPI3) of investigated soil samples.

An independent analysis of the CPI indices was conducted separately in order to assess the biogenic hydrocarbons’ contribution to the OM of these samples. A low contribution of short-chain alkane homologues CPI 1 (*n*-C_10-23_: 9.95–28.73%, Figure 4) and also a low contribution of a full range of *n*-alkanes CPI3 (*n*-C_10-33_: 23.48–40.13%) were found for the soil samples in this study. The highest contribution was found from the mid-chain alkanes CPI2 (*n*-C_24-33_: 37.94–60.11%; Figure 4). These results suggest that both water macrophytes and terrestrial higher plants made a substantial contribution to the OM of the samples analyzed, but also, varying levels of petroleum pollution in these samples are indicated. Reaching its peak at either *n*-C_27_ or *n*-C_29_, the *n*-alkane patterns of the mid-chain *n*-alkanes (range from C_24_ to C_33_, CPI2) exhibited significant odd vs. even prevalence, as indicated by the CPI1 values for the samples that were found to be between 1.71 and 8.00. The results for each of the sampling locations showed a significant presence of mid-chain *n*-alkanes, which is likely due to the contributions of floating and submerged macrophytes. CPI2 values that are close to or less than 1 suggest that the soil samples were mixed with a combination of petroleum and recycled organic matter. Odd and even low-chain *n*-alkanes in the range of *n*-C_24_-_33_ are distributed uniformly (CPI2, Table 2). There is a higher concentration of short-chain *n*-alkanes in the samples Z-9 and Z-19, which is also reflected in the values of the other parameters (Table 2). However, the results for CPI2 show an even carbon numbers prevalence for *n*-alkanes in the *n*-C_24_-_33_ range for some sampling points (Z-2, Z-15, and Z-16, as indicated by the CPI2 values lower than 1; Table 2) and indicate possible petroleum-derived inputs to the OM at these microlocations [44].

The proportion of short to long *n*-alkanes (S/L) is another approach for determination of the *n*-alkane sources in the soil OM. While values of S/L > 2 usually suggest the presence of petroleum compounds in the environment, values of S/L that are ≈ 1 point to the biogenic origin (from phytoplankton’s, algae, higher plants, animals, etc.) of *n*-alkanes. The obtained results for the S/L ratio for the samples analyzed in this study are in the range of 0.05 to 2.26, with an average value of 0.38 ± 0.10. The biogenic sources may be the reason for the values of the S/L ratio being < 1 for almost all samples. The only exemption is the sample taken from the sampling point Z-10 that is close to the Belgrade–Novi Sad Highway (Pan-European corridor X) and has a high value for S/L of 2.26. The CPI results for this sampling point also confirm the notable impact of petroleum compounds (excluding petroleum leaks) is most probably due to the increased population density and heavy traffic. The occurrence of the light *n*-alkanes suggests recent hydrocarbon intake as waste fuels, based on Ines et al. [45]. The impact of both the traffic volume and the number of residents on the soil contamination was also observed earlier in New Belgrade (the preceding work) [19], Madrid [46], and Tehran [47].

**Figure 5:**
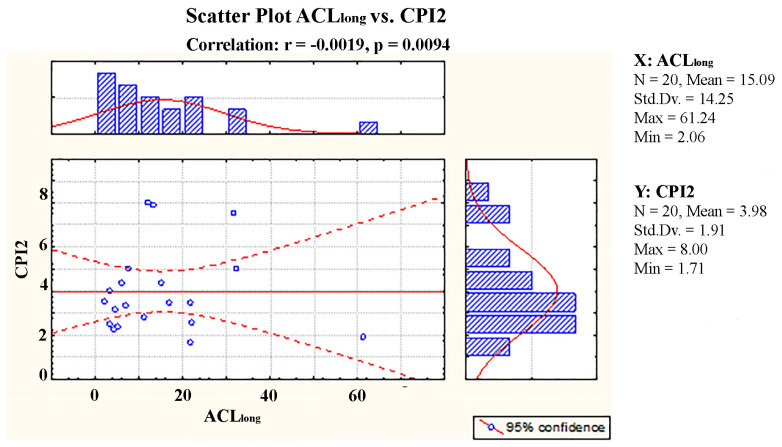
Relationship between the Average Chain Length and the Carbon Preference Index CPI2 in the urban soil samples.

Using the odd carbon numbers of the higher plants *n*-alkanes as a basis, the average chain length (ACL) and alkane index (AI) are two additional tools that are also used for the identification of the source of *n*-alkanes in the environment [48]. The range of ACL values for the soil samples investigated in this study was found to be between 2.06 and 61.24. Furthermore, in addition to the major *n*-alkane contributors previously mentioned in the text, it is probable that the anthropogenic hydrocarbon input is the underlying cause for the low ACL values. Since *n*-alkanes are used to calculate both CPI and ACL, a significant positive correlation between ACL_long_ and terrestrial higher plant *n*-alkanes should be expected if higher plants were the only source of *n*-alkanes in the samples investigated. However, since the samples were contributed with hydrocarbons from various sources, no notable correlation was found between ACL_long_ and CPI2 (*n*-C24-33) (Figure 5).

These results agree well with the results of a previous investigation [49] that indicates how the CPI value illustrates the complex mechanisms of the plant-to-sediment transfer. These processes include the addition of petrogenic hydrocarbons to samples that contain only biogenic hydrocarbons, as well as several admixtures of various vegetative inputs and biodegradation. Vegetation may also influence the soil alkane ACL values. In our study settings, a variety of remains from C_3_ and C_4_ plants are present. We therefore expect the average ACL values to be supported by the AI ratio. The C_4_-type plant footprint is distinguished by greater amounts of long-chain C_31_ and C_33_ *n*-alkanes, whereas the C_3_-type plant footprint is marked by higher levels of long-chain C_27_ and C_29_ alkanes. AI values greater than 0.5 usually occur in vegetation that is dominated by C4 plants, such as grasses and herbs. On the contrary, assemblages of forests exhibit AI values that are lower than 0.5 [50]. Based on the obtained values, C_3_ and C_4_ ecosystems are equally represented in the organic composition of the researched area (Table 2).

The Paq, Pwax, and TAR ratios were used as indicators for more precise determination of biogenic *n*-alkanes. According to Ficken et al. [51], Paq values change depending on the type of plant: 0.0–0.1 for land plants, 0.1–0.4 for large aquatic plants, and 0.4–1.0 for submerged or floating aquatic plants. The Paq ratios in the analyzed samples varied from 0.08 to 0.51, indicating that soil samples had mixed organic matter input from both submerged and terrestrial macrophytes. Samples from sampling points Z-10 and Z-16 that have high Paq values of 0.4 suggest a significant contribution from submerged aquatic plants. This aligns with low ACL values and with high prevalence of short-chain *n*-alkanes. According to Zheng et al. [52], Pwax represents the proportion of waxy hydrocarbons that originate from terrestrial and emergent macrophytes. In this study, the samples were characterized with Pwax ratios from 0.56 to 0.93, which indicates a dominant input from terrestrial macrophytes.

**Figure 6:**
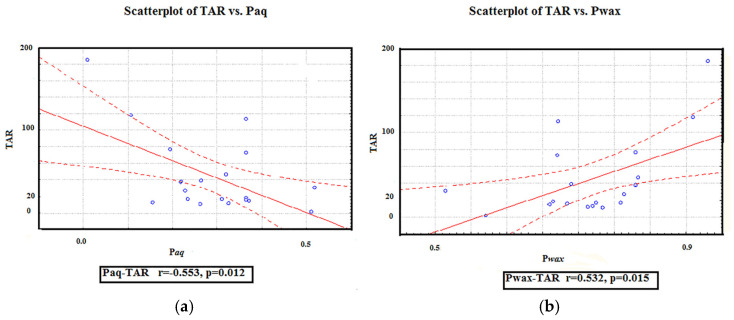
Cross-plots of the terrigenous/aquatic ratio, versus proxy ratios: submerged/floating aquatic macrophyte (Paq) (**a**) and terrestrial plants Pwax (**b**).

The TAR values were calculated to identify possible shifts in the relative contributions of OM, from the aquatic and terrestrial vegetation to the soil environment. The TAR ratio values are very variable (Figure 3), reflecting changes in the relative contributions of aquatic and terrestrial plant-derived organic matter across the sampling area. The ratios of TAR are in the range from 1.31 (Z-10) to 185.11 (Z-9). According to these results, it can be concluded that the terrigenous input to the OM of the samples investigated is predominant, as it can be seen from the negative relationship between TAR and Paq. As expected, TAR is considerably positively correlated with Pwax (Figure 6).

Serving as a geochemical marker for redox conditions during OM deposition in the environment, the Pr/Phy ratio has been frequently used in soil environmental studies. According to Salisu et al. [53], alterations occurring in the phytol side chain of chlorophyll during diagenesis are believed to form pristane in oxygen-rich conditions, while phytane is typically formed in oxygen-poor conditions. Due to the presence of oxygen in the initial phases of the diagenesis of land plants, this procedure usually leads to the formation of decomposed organic material with an elevated Pr/Phy ratio [54]. On the other hand, aquatic ecosystems are more sensitive to the process of anaerobic decomposition, which lowers the Pr/Phy ratio. However, evaluation of the redox conditions in depositional environments using the sedimentary Pr/Phy ratio is a complex task due to the presence of hydrocarbons from various natural sources (e.g., bacteria) and also due to human activities related to petroleum products. Nevertheless, it is generally accepted that values of the Pr/Phy ratio of less than 0.8 suggest reducing saline to hypersaline conditions in the depositional environment; Pr/Phy > 1 corresponds to petroleum pollution, while a Pr/Phy ratio higher than 3 indicates that continental plants were deposited in the environments with oxygen levels ranging from oxic to suboxic [55]. The average value for the ratio Pr/Phy in the analyzed soil samples is 1.04 ± 0.79, which indicates the presence of petroleum contamination and suggests slightly reducing conditions in the depositional environment for a majority of the samples investigated in this study [56]. In general, this ratio has low values in more than 65% of the samples analyzed in this study. However, Pr/Phy ratios lower than 3 in other samples (1.31–2.57) suggest suboxic conditions in the environment during the OM deposition [57]. These results indicate a connection between the organic source inputs and the fluctuation of the redox potential in the depositional environment of the soil samples investigated. According to these results, less oxic conditions are linked to the higher content of aquatic organic matter, which might suggest an occasionally increased water table. Due to the fact that pristane and phytane show greater resistance to biodegradation compared to *n*-alkanes [58], the ratios Pr/*n*-C_17_ and Phy/*n*-C_18_ usually increase significantly beyond 1 in the cases when the OM undergoes extensive biodegradation [59], which is not the case with our samples.

### 3.3. Correlation Matrix

A number of significant correlations (*p* < 0.01) and positive correlations (*p* < 0.05) were obtained in this study (Table 3), suggesting common natural or anthropogenic origins of the OM in the samples.

### 3.4. Q-Mode Cluster Analysis

**Figure 7:**
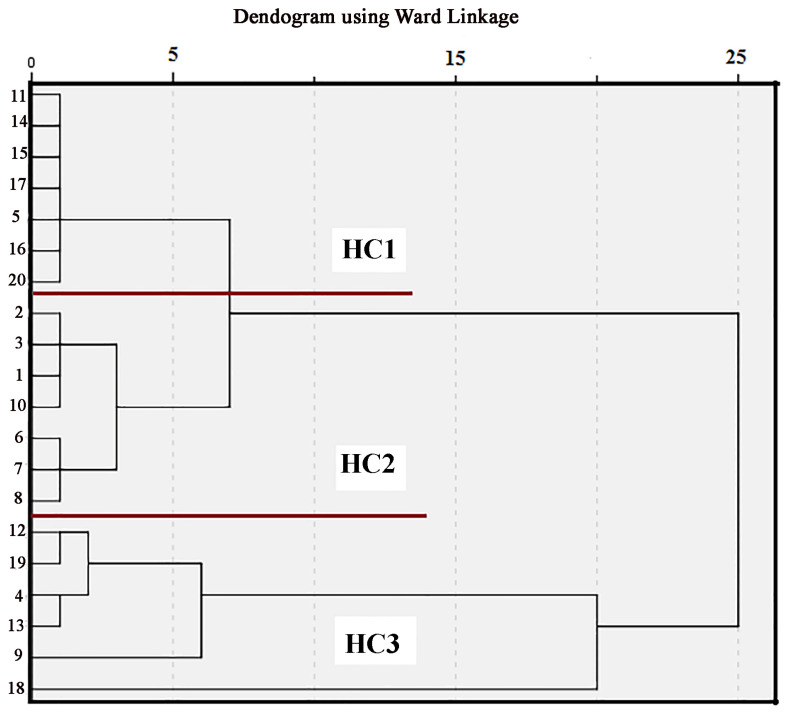
The dendrogram was obtained by applying the Q-mode hierarchical cluster analysis on the evaluation indices calculated on the basis of the distribution of *n*-alkanes in OM of the urban soils analyzed in this study.

Statistical treatment was applied by using the parameters shown in Table 2. The first exploratory method included application of hierarchical cluster analysis (HCA) on the log-transformed data from 20 sampling locations. Spatial HCA found matching monitoring locations and created a dendrogram, clustering all samples into distinct groups. Under squared Euclidean, HCA was applied to the dataset using the Ward method. Three distinct clusters of soil samples have been identified from the results of the Q-mode hierarchical cluster analysis, based on the extent of grouping (Figure 7). Seven samples make the Group HC1 (Z-5, Z-11, Z-14, Z-15, Z-16, Z-17, and Z-20); Group HC2 also consists of seven samples (Z-1, Z-2, Z-3, Z-6, Z-7, Z-8, and Z-10); and group HC3 consists of six samples (Z-4, Z-9, Z-12, Z-13, Z-18 and Z-19). These results confirm that different plants, along with human-made inputs, influenced the composition of the soil organic matter in the investigated location.

Due to a decrease in the biogenic contribution at all locations, the majority of the HC1 cluster can be attributed to the native organic material. C4 plants in soil organic matter created a more powerful signal. At some sampling locations, the HC1 cluster showed increased contributions from aquatic organic matter, as shown by lower TAR index values and higher Paq values. These results could possibly be connected to a periodic increase in the water level in the investigated area. The changes in Pr/Phy levels in this alluvial area suggest the variability of redox potential during the deposition of the sediment. The second cluster, HC2, is defined by the *n*-alkanes predominantly from higher plants and from traffic sources. This group contains more plant compounds compared to the other groups, which follows data from the previously examined diagnostic indices. Alkane compositions found in sedimentary layers, such as in the Z-10 soil sample (which is situated in suburban areas near residential blocks and a moderately busy highway), could be due to the uptake of *n*-alkanes from non-petroleum seep sources. Cluster HC3 samples have the highest levels of TPH, UCM CPI all, TAR, and Pwax indices, higher than in the samples of the other clusters. Contamination in these samples most likely originates from petrogenic sources throughout the history of this area and from a continuous influence of biogenic inputs. The high value of CPI at two sampling points indicates that they were uncontaminated; however, according to the values of the other indices for these sites, they might be affected by both petrogenic and biogenic inputs. The results also confirmed that the proportion of C_3_ and C_4_ plants varied over time in this area.

## 4. Conclusions

This is the first and one of the major investigations of the petroleum pollution of soil in the urban part of New Belgrade, close to the Sava River alluvial area. Several criteria have been employed in the present paper to assess the degree of soil contamination. The majority of evaluated geochemical indices showed that anthropogenic activity has played a major role in soil pollution of the alluvial area in New Belgrade. The *n*-alkanes, as lipid biomarkers, indicated that petroleum was the main source of the low-carbon number in the analyzed soil samples. Because of the high diversity of *n*-alkanes sources, identification of the type of each pollution source in the given area is a challenging task, which was demonstrated by the application of the geochemical indices. Based on the results obtained, it can be inferred that the soil at the analyzed location, in the urban part of New Belgrade, was contaminated from three different sources: petroleum, biogenic (from plants), and traffic. Petroleum pollution, with the potential to impact human health and also endanger the quality of the environment, implies a need for long-time monitoring of AH pollution in the soil environments. Therefore, the present study helps to identify sources of the hydrocarbon contamination in soils, which is crucial for soil conservation efforts. Our future research will focus on optimization of operational parameters to maximize the potential for biodegradation of soil contaminated with petroleum hydrocarbons, with microbial consortia and ecological and health risk assessment impacts on that whole ecosystem.

## Figures and Tables

**Table 1:** The content of the extracted organic substance, aliphatic hydrocarbons, aromatic hydrocarbons, asphaltenes, and polar NSO compounds.

Sample	Extracted Organic Matter (mg/kg)	I Fraction (mg/kg)	II Fraction (mg/kg)	III Fraction (mg/kg)	Range *n*-Alkanes	Max *n*-Alkanes
Z-1	317.23	/	34.95	110.22	C_10-33_	C_20_, C_29_
Z-2	395.23	29.06	46.50	129.81	C_10-33_	C_29_
Z-3	361.80	38.19	48.24	132.66	C_10-33_	C_29_
Z-4	102.59	14.66	3.66	18.32	C_10-33_	C_29_
Z-5	242.01	21.64	37.38	27.55	C_10-33_	C_29_
Z-6	312.30	18.26	38.35	73.05	C_10-33_	C_29_
Z-7	476.08	11.90	71.41	130.92	C_10-33_	C_29_
Z-8	267.14	/	27.07	68.59	C_10-33_	C_27_, C_29_
Z-9	318.17	19.75	24.14	61.44	C_10-33_	C_31_
Z-10	1388.03	8.29	24.86	82.87	C_10-33_	C_20_
Z-11	241.46	31.22	33.31	70.77	C_10-33_	C_31_
Z-12	278.34	/	17.04	58.70	C_10-33_	C_29_
Z-13	431.20	7.70	30.80	128.33	C_10-33_	C_31_
Z-14	344.50	14.76	27.07	54.14	C_10-33_	C_31_
Z-15	678.21	18.63	26.08	89.43	C_10-33_	C_31_
Z-16	1429	12.99	110.39	82.25	C_10-33_	C_22_/_C27_
Z-17	556.42	7.25	18.12	27.19	C_10-33_	C_31_
Z-18	231.80	2.37	18.92	73.32	C_10-33_	C_29_
Z-19	217.13	/	/	60.10	C_10-33_	C_31_
Z-20	167.57	1.90	/	36.18	C_10-33_	C_29_

I fraction: saturated hydrocarbons; II fraction: aromatic hydrocarbons; III fraction: asphaltenes and polar NSO compounds.

**Table 2:** Different evaluation indices calculated on the basis of the distribution of *n*-alkanes and isoprenoids in the organic matter isolated from the analyzed soil samples.

Sample	S/L	ACL	CPI1	CPI2	CPI3	TAR	Paq	Pwax	AI	TPH	UCM	U/R	Pr/Phy	Pr/*n*C_17_	Phy/*n*C_18_
Z-1	0.31	21.90	2.57	1.30	1.98	12.32	0.33	0.76	0.47	70.5	54.80	3.10	0.65	0.89	0.63
Z-2	0.37	6.90	3.38	0.92	2.13	16.75	0.31	0.77	0.31	66.0	70.40	6.50	0.56	0.57	0.54
Z-3	0.32	12.13	8.00	1.33	3.99	16.66	0.23	0.81	0.13	49.6	76.90	5.60	0.32	0.91	0.95
Z-4	0.20	5.00	2.37	1.58	2.00	72.96	0.36	0.70	0.34	67.1	53.8	1.50	2.57	0.78	0.80
Z-5	0.19	11.07	2.84	1.15	2.26	27.33	0.23	0.81	0.79	70.4	3.01	0.18	0.74	0.64	0.63
Z-6	0.18	15.00	4.36	1.28	3.11	37.45	0.22	0.83	0.27	57.1	30.1	0.94	0.73	0.63	0.67
Z-7	0.23	32.29	5.04	1.02	3.12	47.16	0.32	0.83	0.35	59.5	56.7	2.48	0.37	0.49	0.62
Z-8	0.18	61.24	1.94	1.47	1.70	39.72	0.26	0.74	0.46	77.7	42.3	2.08	0.95	0.58	0.70
Z-9	0.05	31.65	7.57	2.33	6.64	185.11	0.08	0.93	0.59	88.0	48.8	3.98	1.31	0.64	0.73
Z-10	2.26	21.71	3.45	1.06	1.39	1.315	0.51	0.62	0.37	77.5	78.4	3.70	0.10	0.52	0.65
Z-11	0.29	7.51	5.03	1.22	3.24	12.70	0.15	0.77	0.52	79.6	20.2	0.83	0.26	0.77	0.98
Z-12	0.25	2.06	3.51	1.16	2.33	113.82	0.36	0.72	0.44	77.0	2.9	0.73	1.41	0.64	0.78
Z-13	0.22	6.00	4.37	1.89	3.38	76.82	0.19	0.83	0.56	78.4	12.2	3.75	1.90	0.60	0.56
Z-14	0.51	3.18	4.01	1.75	2.67	11.56	0.26	0.79	0.51	79.5	21.4	3.28	1.18	0.68	0.59
Z-15	0.43	4.30	2.30	0.93	1.58	18.54	0.36	0.71	0.50	70.9	16.6	2.25	1.60	0.72	0.78
Z-16	0.68	3.28	2.51	0.79	1.28	30.92	0.51	0.56	0.28	77.9	7.7	1.00	2.15	0.77	0.70
Z-17	0.35	21.80	1.71	1.24	1.45	14.57	0.37	0.71	0.85	75.3	17.0	1.38	1.76	0.65	0.49
Z-18	0.19	16.96	3.46	1.32	2.64	37.94	0.22	0.83	0.45	244	33.7	2.48	1.10	0.12	0.80
Z-19	0.13	13.31	7.92	2.21	6.01	118.05	0.11	0.91	0.58	109	17.2	1.80	2.18	0.63	0.49
Z-20	0.32	4.40	3.17	1.26	2.26	15.57	0.36	0.73	0.24	102	18.0	1.30	0.05	0.39	0.63

S/L—The ratio of short to long *n*-alkanes; ACL: average chain length; CPI1: short-chain alkane homologues (*n*-C10-23); CPI2: mid-chain alkanes (*n*-C24-33); CPI3: full distribution of *n*-alkanes (*n*-C10-33); TAR: the terrigenous/aquatic ratio; Proxy ratios (Paq and Pwax); AI: Alkane Index; TPH: total petroleum hydrocarbons; UCM: unresolved complex mixture; U/R: the ratio of GC-unresolved and GC-resolved HAs; Pr/Phy: pristane/phytane ratio; Pr/*n*-C_17_ and Phy/*n*-C_18_: isoprenoids/*n*-alkanes.

**Table 3:** Results of Pearson correlation analysis.

	S/L	CPI2	CPI1	CPI3	AI	ACL	TAR	Pwax	Paq	Phy/C_18_	Pr/C_17_	Pr/Phy	TPH	UCM	U/R
**S/L**															
**CPI2**	−0.18														
**CPI1**	−0.33	0.58 *													
**CPI3**	−0.38	0.91 **	0.79*												
**AI**	−0.17	−0.16	0.3	0.09											
**ACL**	−0.09	0.06	−0.41	0.06	0.19										
**TAR**	−0.39	0.51 *	0.70 *	0.73 **	0.2	−0.58 *									
**Pwax**	−0.62 **	0.68 *	0.69 *	0.83 **	0.32	−0.3	0.89								
**Paq**	0.68 **	−0.65 *	−0.71 **	−0.81 **	−0.29	0.33	−0.49	−0.92 **							
**Phy/C_18_**	−0.05	0.12	0.02	0.03	−0.02	−0.05	−0.03	−0.16							
**Pr/C_17_**	−0.06	0.18	−0.17	0.03	−0.37	−0.13	−0.03	−0.09	0.03						
**Pr/Phy**	−0.02	−0.08	0.35	0.09	0.3	−0.03	0.45	0.09	0.39	0.48					
**TPH**	−0.09	−0.14	−0.13	0.09	0.1	0.04	0.09	0.19	−0.55	0.78 *	0.013				
**UCM**	0.35	0.23	−0.35	0.06	−0.49	−0.13	0.14	0.04	0.07	−070 **	−0.16	−0.6	−0.15		
**U/R**	0.18	0.18	0.13	0.21	−0.33	−0.32	−0.03	0.2	−0.09	−0.23	−0.08	0.2	−0.09	0.72 **	

S/L: The proportion of short to long *n*-alkanes, ACL: Average Chain Length, CPI1: short-chain alkane homologs, CPI2: mid-chain alkanes, CPI3: full distribution of *n*-alkanes, TAR: The terrigenous/aquatic ratio, Proxy ratios (Paq and Pwax), AI: Alkane Index, TPH: Total petroleum hydrocarbons, Unresolved Complex Mixture (UCM), U/R- Unresolved/Resolved ratio, Pristane/Phytane Ratio (Pr/Phy), Isoprenoids/*n*-alkanes (Pr/*n*-C_17_ and Ph/*n*-C_18_). * Correlation is significant at the 0.05 level. ** Correlation is significant at the 0.01 level.

## Data Availability

The authors declare that all data generated or analyzed during this study are included in the published article.

## Supplementary Materials

The following supporting information can be downloaded at https://www.mdpi.com/article/10.3390/molecules30010154/s1: Supplemental material was listed below including: 1. Soil sampling and instrumental analysis, 2. The organic geochemical indices and 3. References. Refs: [60,61,62,63,64,65,66] are also cited in Supplementary Materials.
