# Supplementary material for: Lipid Biomarkers in Urban Soils of the Alluvial Area near Sava River, Belgrade, Serbia
Source: Molecules. 2025 Jan 3;30(1):154. doi: 10.3390/molecules30010154 (PMC11721175; doi:10.3390/molecules30010154)
Supplement: Supplementary file 1 [file molecules-30-00154-s001.zip › molecules-3345329-supplementary.pdf]

Lipid Biomarkers in Urban Soils of the Alluvial Area near Sava River, Belgrade, Serbia

Gordana Dević [1,*], Sandra Bulatović [1], Jelena Avdalović [1], Nenad Marić [2], Jelena Milić [1], Mila Ilić [1] and Tatjana Šolević Knudsen [1]

[1] Institute of Chemistry, Technology and Metallurgy, National Institute of the Republic of Serbia, University of Belgrade, Njegoševa 12, 11000 Belgrade, Serbia; sandra.bulatovic@ihtm.bg.ac.rs (S.B.); javdalovic@chem.bg.ac.rs (J.A.); jelenamilic@chem.bg.ac.rs (J.M.); milailic@chem.bg.ac.rs (M.I.); tsolevic@chem.bg.ac.rs (T.Š.K.)

[2] Faculty of Forestry, University of Belgrade, Kneza Višeslava 1, 11030 Belgrade, Serbia; nenad.maric@sfb.bg.ac.rs

* Correspondence: gordanadevic@gmail.com

Supplemental material listed below includes:

1. **Soil sampling and instrumental analysis**

2. **The organic geochemical indices**

3. **References**

1. Soil sampling and instrumental analysis

In May 2019 the soil samples were collected from 20 sampling micro-locations (sites) at depths 0.0–0.3 m (Fig.1). 1 kg of each soil sample was taken using a cup type auger. The samples were placed in aluminum bags, and kept at 4 °C in the mini fridge, until transferred to the laboratory for analyses. Before sampling, all equipment was washed and rinsed with methanol and deionized water, to avoid potential contamination risks. For determination of the Aliphatic hydrocarbons (AHs), the content of Total Petroleum Hydrocarbons (TPH) was previously determined. TPH was determined according to the standard methods ISO 16703, and DIN EN 14345 [1-2].

The total solvent extractable organic substance from the analyzed soil samples was extracted for 36 h with dichloromethane as a solvent, using a Soxhlet method. The isolated extracts were then concentrated on a rotary evaporator and completely dried under stream of N_2. After drying, the concentration of the organic substance of each sample was gravimetrically determined (Table 1). The samples were then saponified

overnight, using a 5 % solution of KOH in methanol. The neutralization of the samples was provided with the addition of 10 % HCL. The extracts of the organic substance of all soil samples were dissolved in *n*-hexane, and fractionated by liquid chromatography into saturated hydrocarbons (Fraction I), aromatic hydrocarbons (Fraction II), and polar compounds: alcohols and keto compounds (Fraction III), using a column with alumina and silica gel (1 cm x 20 cm). Aluminum oxide and silica gel were previously activated by heating at 180°C at 24h. Fraction I was eluted with *n*-hexane, Fraction II with dichloromethane, and Fraction III with a mixture of dichloromethane and methanol (1:1). Each fraction was concentrated to 1 ml and evaporated until completely dried, under a stream of N_2 stream. After drying, masses of each fraction of all samples were gravimetric determined (Table 1). For identification of *n*-alkanes and isoprenoids, as lipid biomarkers, dried samples of Fraction I were re-dissolved in *n*-hexane and analyzed using Gas Chromatography with a Flame Ionization Detector (GC-FID). The GC-FID analysis was carried out on an Agilent 7890A gas chromatograph with flame ionization detector (Agilent, Santa Clara, CA, United States)" equipped with a TG-5MT capillary column (30 m x 0.25 mm, 0.25 µm film thickness, Agilent, Santa Clara, CA, United States).

The column was heated from 80–330 °C (10 °C / min). Chromatographic conditions were: 1 µL splitless injection at 60 °C oven temperature (injector temperature 270 °C, splitless time 60 s), 3 min of holding the starting temperature, and a programmed temperature increase of 3 °C / min to 300 °C. The hydrogen carrier gas flow was 1 ml/min.

All the standards for *n*-alkanes were purchased from Merck (Germany), including all solvents and chemicals used for the performed analyses.

For the QA/QC control, a blank soil has been traditionally prepared and used in our lab. For that purpose, 5 kg of the surface layer of the Danube River bottom sediment was collected. The sediment was precleaned to remove any large biogenic, mineral or other contents, and dried in an oven set at 130°C for four days. Dry sediment was sieved to collect the fraction with a diameter of less than 2 mm. This sediment fraction was cleaned through several steps of extractions. For that purpose, the sediment was extracted for 36 h using a Soxhlet method, first with hexane as a solvent, than with dichloromethane, and finally with methanol. After each Soxhlet extraction, the remaining solvent in the solid phase was removed by centrifugation, and the solid

phase was dried in an oven set at 130°C for four days. After the final drying step, the solid material was checked for cleanliness to confirm that it was free from petroleum hydrocarbons (TPH). After the quality check, this material was transferred to a clean air tight glass container, labeled as "soil blank for TPH", and kept in a cool, dry place away from sources of contamination. This blank soil was used in the TPH analysis as a control sample to validate that any detected petroleum hydrocarbons in our environmental samples were not due to contamination from the soil or preparation process.

2. The organic geochemical indices

Based on the distribution and abundance of *n*-alkanes and isoprenoids, several indices were used in this study to identify the origin of ACs.

Carbon Preference Index (CPI) is index widely used to differentiate natural and anthropogenic sources of organic matter, and it can be calculated using Equation (1) [3]:

$$CPI_{(24-33)} = 0.5 \times \left[\frac{\Sigma(Cn\ odd)_{25-33}}{\Sigma(Cn\ even)_{24-32}} + \frac{\Sigma(Cn\ odd)_{25-33}}{\Sigma(Cn\ even)_{26-32}}\right] \quad (1)$$

where C_n *odd* were odd-numbered, and C_n *even* were even-numbered C hydrocarbon chains.

The ratio of short to long hydrocarbons chains (S/L) was calculated to determine the dominant bacteria, algae or phytoplankton-derived and/or higher plant-derived organic matter, according to Equation (2) [3]:

$$S/L = \Sigma(C_n)_{10-23}/\Sigma(C_n)_{24-33} \quad (2)$$

where C_n is the relative abundance of each *n*-alkane from C_{10} to C_{33}.

Average Chain Length (ACL) values of *n*-alkanes can be used to show the distribution of C_3 and C_4 plants [3, 4], and describes the average number of carbon atoms per molecule based on the abundance of the odd-carbon-numbered higher plant *n*-alkanes. The ACL was calculated using the following Equation (3):

$$ACL_{long} = \Sigma(n \times C_n)_{24-33}/\Sigma(C_n)_{24-33} \quad (3)$$

where n is a number of C atoms, and C_n is the relative abundance of each n-alkane from C_{10} to C_{33}.

Values of Alkane Index (AI) higher than 0.5 indicate an influence of grasses (C4 plants) as organic carbon sources [5]. On the other hand, AI values lower than 0.5 indicate that the terrestrial organic matter is derived from C_3 plants [6]. The AI was calculated using the Equation (4):

$$AI = {}^{nC_{31}}/_{nC_{31}} + nC_{29} \tag{4}$$

Proxy ratios (Paq and Pwax*)* reflect the submerged/floating aquatic macrophyte input to organic matter relative to that from the terrestrial plants and vice versa. Odd-numbered mid-chain n-alkanes including C_{23} and C_{25} are abundant in submerged and emerged vegetation, while C_{29} and C_{31} are abundant in terrestrial plants. The Paq and Pwax was calculated using the Equations (5) and (6) [7]:

$$P_{aq} = \frac{(C_{23} + C_{25})}{(C_{23}+C_{25}+C_{27}+C_{31})} \tag{5}$$

$$P_{wax} = \frac{(C_{27} + C_{29}+C_{31})}{(C_{23}+C_{25}+C_{27}+C_{29}+C_{31})} \tag{6}$$

The terrigenous/aquatic ratio (TAR) assumes that photosynthetic algae and bacteria are characterized by short-chain n-alkanes (C_{17}) when compared with the C_{27}, C_{29}, and C_{31}-rich molecules present in vascular plants [8]. The TAR ratio can be used as a qualitative proxy for the relative contributions of organic matter from terrestrial areas and aquatic sources. The TAR was calculated using the Equation (7):

$$TAR = \frac{(C_{27} + C_{29}+C_{31})}{(C_{15} + C_{17}+C_{19})} \tag{7}$$

Pristane/Phytane Ratio (Pr/Phy) ratio is a parameter usually used to identify the organic matter source. Pristane and phytane are constituents in petroleum and in environmental studies are target compounds for petroleum spill detection. AHs originating from terrestrial plants contain high a Pr/Phy ratio, above 3.0, while lower values point to petroleum pollution [9]. Isoprenoids/n-alkanes (Pr/n-C_{17} and Phy/n-C_{18}) ratios provide valuable information on biodegradation [10].

3. References:

1. Avdalović, J; Đurić, A.; Miletić, S.; Ilić M.; Milić J.; Vrvić, M. M. Treatment of a mud pit by bioremediation. *Waste Manag. Res*. **2016**, 34, 734–739. https://doi.org/10.1177/0734242X16652961
2. Beškoski, P. V.; Gojgić-Cvijović, G.; Milić, J.; Ilić, M.; Miletić, S.; Šolević, T.; Vrvić, M. M. *Ex situ* bioremediation of a soil contaminated by mazut (heavy residual fuel oil) – a field experiment. *Chemosphere* **2011**, 8, 34–40. https://doi.org/10.1016/j.chemosphere.2011.01.020
3. Kumar, M., Boski, T., González-Vila, F. J., de la Rosa, J. M., González-Pérez, José A., Discerning natural and anthropogenic organic matter inputs to salt marsh sediments of Ria Formosa lagoon (South Portugal). *Environ. Sci. Poll. Res.* **2020**, 27, 28962–28985. https://doi.org/10.1007/s11356-020-09235-9
4. Vogts, A.; Moossen, H.; Rommerskirchen, F.; Rullkötter, J. Distribution patterns and stable carbon isotopic composition of alkanes and alkan-1-ols from plant waxes of African rain forest and savanna C_3 species. *Org. Geochem*. **2009,** 40, 1037–1054. https://doi.org/10.1016/j.orggeochem.2009.07.011
5. Zhang, Z.; Zhao, M;, Eglinton, G.; Lu, H.; Huang, C.Y. Leaf wax lipids as paleovegetational and paleoenvironmental proxies for the Chinese Loess Plateau over the last 170kyr. *Quat. Sci. Rev.* **2006**, 25, 575–594. https://doi.org/10.1016/j.quascirev.2005.03.009
6. Rommerskirchen, F.; Plader, A.; Eglinton, G.; Chikaraishi, Y.; Rullkotter, J. Chemotaxonomic significance of distribution and stable carbon isotopic composition of long-chain alkanes and alkan-1-ols in C_4 grass waxes. *Org. Geochem*. **2006,** 37, 1303–1332. https://doi.org/10.1016/j.orggeochem.2005.12.013
7. Cheng, X.; Hou, D.; Zhou, X.; Liu, J.; Diao, H.; Wei L. Geochemical Characterization of the Eocene Coal-Bearing Source Rocks, Xihu Sag, East China Sea Shelf Basin, China: Implications for Origin and Depositional Environment of Organic Matter and Hydrocarbon Potential. *Minerals*, **2021**, 11, 909. https://doi.org/10.3390/min11080909
8. Bourbonniere, R. A.; Meyers, P. A. Sedimentary geolipid records of historical changes in the watersheds and productivities of Lakes Ontario and Erie. Limnol. Oceanogr. **1996,** 41, 352-359. https://doi.org/10.4319/lo.1996.41.2.0352
9. Oraegbunam, C.; Osuji, L.; Onojake, M.; Abrakasa, S. Geochemical Appraisal of the Depositional Environment and Source Organic Matter of Crude Oils from

Some Oil Fields in Bayelsa State, Nigeria. *Iran. J. Oil Gas Sci. Technol.* **2020,** 9, 1–10. https://doi:10.22050/ijogst.2020.221306.1538

10. Peters, K. E.; Walters, C. C.; Moldowan, J. M. The Biomarker Guide Vol. 1: Biomarkers and Isotopes in the Environment and Human History., **2005,** 2nd edition, Cambridge University Press, New York.
